# Preparation and in-vitro evaluation of indomethacin nanoparticles

**Published:** 2010

**Authors:** A. Rezaei Mokarram, A. Kebriaee zadeh, M. Keshavarz, A. Ahmadi, B. Mohtat

**Affiliations:** 1 Department of formulation and packaging of human biological products, Razi vaccine & serum research institute, Karaj; 2 Department of Toxicology, School of pharmacy, Tehran university of medical sciences, Tehran; 3 Department of Chemistry, faculty of science, Islamic azad university, Karaj branch, Karaj, Iran

**Keywords:** Controlled precipitation, Nano-solid suspension, Indomethacin, Polyvinyl pyrrolidone

## Abstract

**Background and the purpose of the study:**

During the last two decades one of the most important problems in drug formulations has been low aqueous solubility of new molecules. However, numerous techniques, such as milling, co-solvent solubilization and solid dispersion have been used conventionally for aqueous solubility enhancement and the rate of solubility. Recently, nanoparticle engineering processes have been developed and reported for pharmaceutical applications to increase the dissolution rate of low-soluble drugs which in turn may leads to substantial increases in bioavailability. In this study, a controlled precipitation method was used to produce indomethacin nano-solid suspension in a polymeric matrix (as a model), in order to increase the solubility and rate of the dissolution of poorly soluble model drug.

**Methods:**

Nano-solid suspension of indomethacin in polyvinyl pyrrolidine (PVP) was prepared by controlled precipitation technique, characterized by differential scanning calorimetry (DSC), X-ray diffraction (XRD), Fourier Transform Infrared Spectroscopy (FTIR) and evaluated for in vitro solubility and dissolution rate.

**Results and major conclusion:**

Absence of thermal and diffractional peaks in DSC and XRD studies indicated that indomethacin interacts with PVP in solid phase. The solubility of indomethacin in nano-solid suspension compared to crystalline form was increased to about four-fold. It was found that particle size distribution depend to the polymer MW and drug: polymer ratios. Spectroscopy methods and Transmission Electron Microscopy (TEM) images showed that indomethacin dispersed as amorphous nanosize particles in freeze dried powder. Enhanced solubility and dissolution rate of indomethacin compared to physical mixtures and crystalline form of indomethacin (polymorph I), demonstrated that it interacts with PVP via hydrogen bond and probably forming eutectic mixture.

## INTRODUCTION

During the last two decades one of the problems raised in pharmaceutical technology has been poor aqueous solubility and/or dissolution rate of the new molecules (1). Pharmaceutical scientists are constantly seeking new approaches in order to obtain an adequate oral bioavailability. Recently, the formulation of drugs as nanoscale systems (which have a size below 1µm) has rapidly evolved as a drug delivery strategy (2). The major characteristic of these systems is the rapid dissolution rate, which enhance bioavailability after oral administration (3). Current techniques used to obtain drug nanoparticles can be divided into two categories: The first comprises the bottom-up processes that build up particles from dissolved drug molecules (e.g. aerosol flow reactor method) (4), microemulsion template methodology (5), supercritical fluid-based technologies (6), high gravity reactive precipitation (7), controlled precipitation (8) and the melt emulsification method (9). The second category consists of top-down processes that are based on breaking down larger particles e.g. media milling (2) and high-pressure homogenization (10). Typically, these production processes are conducted in liquid, hence forming a nanosuspension. As the total surface area of the resulting nanosuspension particles is typically several orders of magnitude larger compared to a coarse suspension, large quantities of additives may be necessary to ensure adequate stabilization. Although, all marketed products, currently are produced by so-called top-down techniques, in which the nanoparticles are obtained through size reduction into the submicron-range, bottom-up techniques and especially controlled precipitation method, are methods of interest for nanozation of poorly soluble drugs. In this method without any harsh conditions and only with simple equipments one could reduce the particle size to few hundred nanometers range. Therefore, whatever method which is used for the production of nanosuspensions, a careful evaluation of the type and concentration of the stabilizer is a critical stage for the successful production of nanosuspensions. Both polymeric and surfactant stabilizers can be used for this purpose (11).

Indomethacin is a non-steroidal, anti-inflammatory drug (NSAID) with anti-pyretic and analgesic properties. It is categorized as Class II drug that has poor solubility and high permeability. Some studies have shown that the solubility and dissolution rate of poorly soluble drugs, such as indomethacin is enhanced, in combination with high soluble excipeints as solid dispersion (12, 13). In the previous study it was shown that the dispersion of indomethacin co- percipitate with Polyvinyl Pyrrolidone (PVP) is in the colloidal form and the samples were stable for a long lasting time (14).

The aim of this study was, firstly to investigate the feasibility of preparation of indomethacin copercipitation, as vehicle for enhancement of wettability and decreasing agglomerative properties of indomethacin nanoparticle, and finally to investigate the solubility and dissolution rate of the model drug.

## MATERIAL AND METHOD

### 

#### Materials

Indomethacin, [1-(*p*-chlorobenzoyl)-5-methoxy- 2-methylindole-3-acetic acid], as the γ crystal form was kindly gifted by Behdasht Kar Co., (Tehran-Iran). The α-polymorph of indomethacin was prepared by dissolving the γ crystal form in boiling methanol and precipitated with water at room temperature as reported by Kaneniwa *et al.* (15). Purity of two polymorphs was determined by wide-angle X-ray scattering pattern. Polyvinyl Pyrrolidone (PVP) K-25, K-40 and K-360 were purchased from Sigma-Aldreich, (USA). All other reagents were analytical grades and purchased from Merk Co., (Germany). Ultrapure water (Millipore, Direct Q^TM^, France) was used throughout the study.

#### Methods

##### Preparation of IND nanoparticles

Indomethacin nano-solid suspensions were prepared by control pH precipitation followed by freeze dried technique. Briefly, co-percipitates of indomethacin- PVP were prepared over the range of 10- 95% w/w PVP (1:9 to 19:1 polymer-drug ratio). Different ratios of indomethacin and PVP were dissolved in ultra-pure water by adding 20 µl of 1M NaOH, and then the samples passed through membrane filter (0.2 µm). The particles were formed spontaneously after decreasing the pH to 5.5-6 by 1M HCl solution. The nano-solid suspensions prepared, was cooled rapidly to −70 °C and stored overnight. Freeze-drying of the samples was performed with a Telstar freeze-dryer (Terrassa, Spain) at a shelf temperature of −50 °C with a pressure below 1 mbar, then the vials were removed after 48 hrs of drying. The samples were stored in well closed glass container at −20 °C for more investigations. Previous studies have shown that pure amorphous indomethacin in this temperature is physically stable for at least 6 months (16)

##### Particle size distribution

Particle size distribution after re-dispersion of the freeze-dried samples was measured with dynamic photon correlation spectroscopy technique (PCS) using a Zetasizer SZ3000 (malvern instrument, worcestershier, UK.). All the presented data are the mean value of three independent samples produced under identical production conditions.The morphology of the nanoparticles was examined by Transmission Electron-Microscopy (CM 12 Philips, Eindhoven, Netherlands).

##### X-ray powder diffraction

Samples of each indomethacin-PVP co-percipitate were placed into tightly sealed vials and stored at −20 °C. The extent of any physical change or crystallization over a 20-days period was determined. The X-ray data were collected with a SEIFERT-3003 PTS X-ray generator with Cu rotating anode (Germany), 40 kW, 30 mA and scanning speed 4°.

##### Fourier Transform Infrared Spectroscopy (FT-IR)

The fourier transform infrared spectroscopy (FT-IR) spectra were obtained using FT-IR Shimadzu (FT-IR prestige 21). The samples (indomethacin, PVP, physical mixtures or nano-solid dispersions) were grounded and mixed thoroughly with potassium bromide, at 1:5 (sample:potassium bromide) weight ratio. The potassium bromide discs were prepared by compressing the powders at a pressure of 5 tones for 5 min in a hydraulic press. Scans were obtained at a resolution of 2 cm-1, from 2000 to 500 cm’^−1^.

##### Differential Scanning Calorimetry (DSC)

Thermal characteristics of the pure materials, the physical mixtures and nano-solid suspensions were determined by an automatic thermal analyzer system (Shimadzu, DSC- 60). Accurately weighed samples (5–8 mg) were placed in nonhermetically aluminium pans and heated against an empty aluminium pan as a reference. Heating rate was 5 °C/min and nitrogen purge 20 ml/min.

##### Determination of indomethacin content in lyophilized samples

The indomethacin content in samples was analysed by dissolving 10 mg of samples in 10 ml of 0.1 M methanolic HCl (USP). The samples were stirred on a magnetic stirrer at 100 rpm at room temperature for one hour, filtered (0.22 µm, millipore®) and diluted with above medium. The amount of drug was determined spectrophotometrically (Jenway 6505, Dae II Science Co., South Korea) at 259 nm against 0.1 M methanolic HCl as blank.

##### Determination of indomethacin solubility

All the solubility experiments were carried out using the analytical isothermal shake-flask method. The aqueous solubility of indomethacin was measured at25 °C. Saturated solutions were prepared by mixing an excess of solid solute and 20 ml of water into a constant-temperature jacketed glass cells and stirring for 2 days, filtered (cut off, 0.22 µm, millipore®) and the content of dissolved indomethacin was analysed spectrophotometrically at 259 nm.

##### In-vitro release study

Ten milligrams of lyophilized samples containing indomethacin and PVP were suspended in 300 ml of 0.1 M HCl to maintain sink conditions and stirred on a magnetic stirrer at 50 rpm (Kavosh Dissolution Tester, Tehran, Iran) at room temperature. At pre- determined time intervals, 5 ml aliquots were withdrawn, filtered (cut off 0.22 µm) and the amount of dissolved indomethacin was determined spectrophotometrically. A physical mixture containing 10 mg of micronized indomethacin and PVP in 300 ml of 0.1 M HCl was used as reference dispersion for the dissolution test. Each dissolution experiment was performed in triplicates and the average values and standard deviations were calculated.

##### Statistical analyses

Effect of MW and drug ratio on particle size distribution and dissolution rate was determined using unpaired student's t-test. Significance was tested at the 0.05 level of probability. Statistical analysis was performed with the software package SPSS® 11.

## RESULTS AND DISCUSSION

Some researchers have previously shown that numerous polymers such as PVP can improve the solubility and dissolution rate of low soluble drugs (13). Moreover, it has been reported that PVP has an inhibitory effect on re-crystallization rate of indomethacin in amorphous solid dispersions (17). In addition, it has been shown that the crystallization rate of indomethacin is dependent on its molecular mobility in solid dispersion phase (18). Therefore, it has been suggested that PVP can interact with indomethacin via hydrogen bond and consequently prevent re-arrangement of drug molecules to crystalline form. In the previous report it has been shown that indomethacin dispersion within polymeric matrix in solid phase was colloidal form (14). So, it is logical to accept that this polymer can entrap particulated indomethacin at nano-range and in amorphous state for a long lasting time.Based on the above comments, it was found important to investigate whether polymer molecular weight and drug ratio have any effect on particle size distribution and stability of indomethacin nano-solid suspensions.

### 

#### Preparation of polymorph II of indomethacin

**Figure 1:**
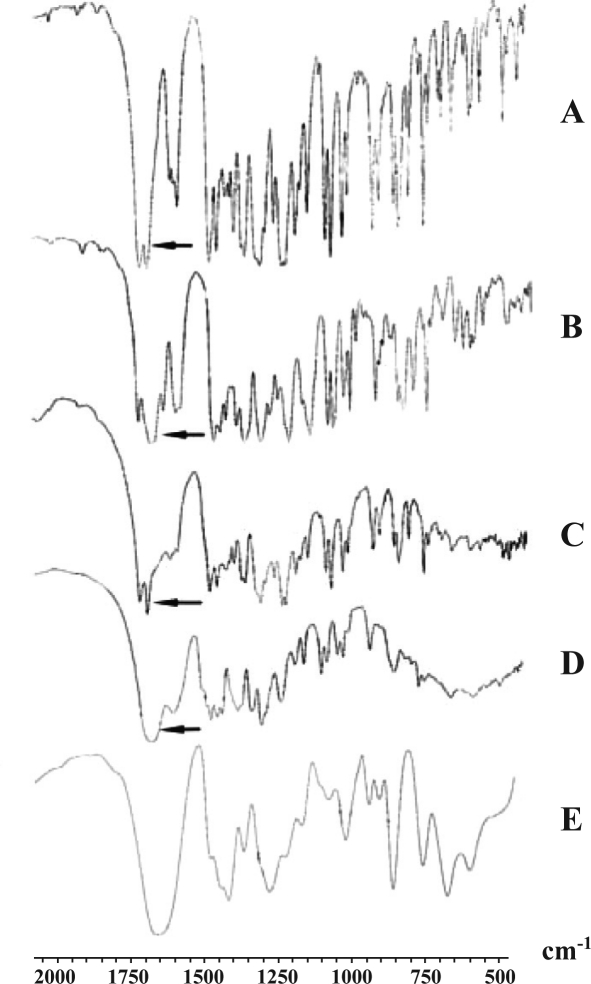
FT-IR pattern of A) IND (polymorph I), B) IND (polymorph II), C) IND:PVP (1:2) physical mixture, D) IND:PVP (1:2) nano-solid suspension and E) pure PVP.

**Figure 2:**
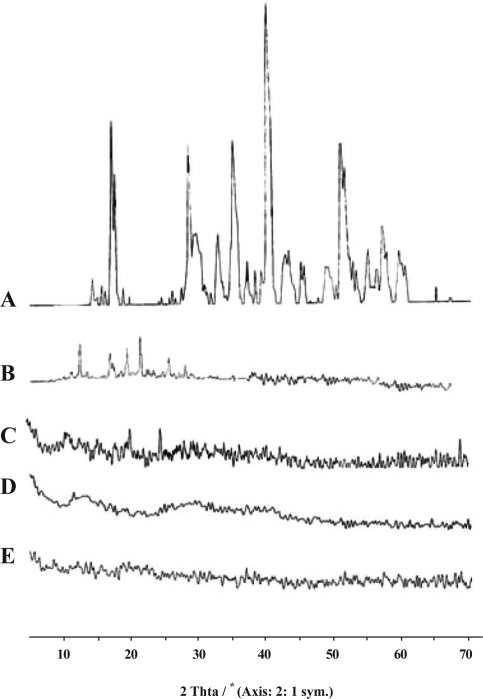
Powder X-ray diffraction pattern of IND:PVP nanosuspensions samples: A) IND (polymorph I), B) IND (polymorph II), C) IND:PVP (2:1) physical mixture, D) IND:PVP (2:1) nanosuspension and E) PVP

Indomethacin is known to exist in more than one non-solvated crystalline form. Most investigations refer to these polymorphs as forms I and II. Form I is the highest melting and lowest solubility polymorph and therefore, it is the thermodynamically stable crystalline form of indomethacin. During the preparation of indomethacin nano-solid suspension, formation of metastable form of crystalline indomethacin (form II) is probable. In order to investigate the physical properties of this polymorph, it was prepared according to Kaneniwa method (15). FT-IR and X-ray diffraction pattern of these polymorphs are shown in figures 1 and 2.

#### Effect of polymer MW and drug ratio on particle size

In order to assess the effect of polymer type on particle size and stability of indomethacin nano-solid suspension, three types of PVP were employed. The particle size range of dispersed indomethacin within polymeric matrix is shown in table 1. These data which are in agreement with previous report (19) show that the nature of the polymers used in solid dispersions has a minor effect on the stability behavior of nano-solid dispersion systems. In other word, the ratios of drug: polymer and capability of the polymer binding to indomethacin molecules are the most important factors which determine indomethacin crystallinity or amorphous states in solid phase (18). This is a logical result, because rate of the crystallization of drug from amorphous state is known to be related to molecular mobility in solid phase (20. Therefore, a high molecular weight polymer with a high glass transition temperature (Tg) will raise the viscoelastic properties of the mixture and decrease the mobility of dispersed drug relative to drug alone and consequently reduce the tendency for crystallization. These results are in agreement with pervious finding (21, 22), in which it has been reported that PVP has an inhibitory effect on amorphous nanoparticle re-crystallization during freeze-drying cycles.

**Table 1:** Effect of polymer MW and drug ratio on the particle size and polydispersity index of selected nano-solid suspension formulation.

Type system	Intrinsic solubility*	Relative solubility**
Nano-solid suspension	5.86±1.2	6.45
Physical mixture	2.30±0.51	2.53
Polymorph I	0.91±0.26	1.00
Polymorph II	1.44±0.34	1.59

PI= polydispersity index

(n = 3)

**Figure 3:**
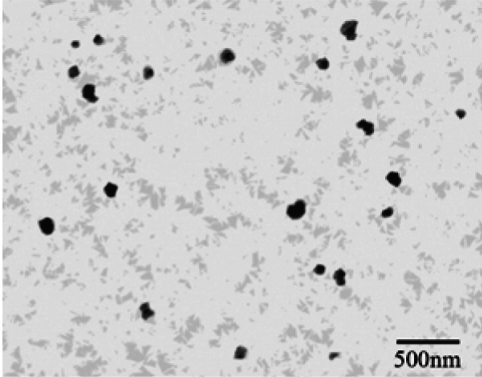
TEM micrograph of indomethacin nanoparticles released from freeze-dried nano-solid suspension (9:1) drug:polymer ratio. Ten µl of medium was taken immediately after dissolving the freeze-dried sample in ultrapure water.

Particle size and TEM micrograph of indomethacin nanoparticles, after re-dispersion of the freeze-dried samples, was measured by DLS technique (Table 1) and TEM (Figure 3) respectively. Base on the results, it seems that mean of the particle size of indomethacin nano-solid suspension increase as polymer MW and drug ratio increased. Furthermore, the polydispersity index (PI) values, which define the particle size distribution, indicated that the samples prepared with high MW polymer (PVP-360) and have low drug contents are more homogenous t h a n t h o s e prepared with low MW polymer (PVP-10) and high drug ratio. This result indicates that indomethacin interactions with PVP-10 are higher than with PVP-360, but lower viscoelasticity property of PVP-10 than PVP-40 and PVP-360 can not prevent aggregation of re-dispersed nanoparticles with each other effectively. The results confirm that the protective effect of PVP on nanoparticle agglomeration is affected by the drug ratio and polymer MW. Previous study has shown that the amount of interactions of indomethacin-PVP increased by decrease of PVP molecular weight. The greatest extent of interactions was found in the solid dispersion prepared with PVP K10 (17).

#### FT-IR analysis

Figure. 2 shows the FT-IR spectra of indomethacin (polymorph I & II), PVP, physical mixture and nano- solid suspension system (1:2). The pattern of C = O stretch vibrations is typically observed in the range of 1600-1750 cm-1. The indomethacin molecule contains two carbonyl groups of the benzoyl and carboxyl moieties. It was suggested that the peaks present in this range must be attributed to the asymmetric benzoyl stretching, while the peak at 1692 cm-1 is assigned to benzoyl vibration (20). In the form II and amorphous indomethacin, the benzoyl vibration occurs at a lower wave number (1688 and 1684 cm-1 respectively). Three other peaks of lower intensity are observed at lower frequencies: 1580, 1590 and 1621 cm-1. PVP shows only a broad and rounded peak (centered at 1666 cm-1) in this range, which it is attributed to the amide carbonyl of the PVP units. The three types of PVP, which were employed to prepare solid dispersions, K10, K40 and PVP K360, differing in MW, cannot be distinguished by IR spectra, because the monomers have identical backbone, differing only in chain length.

All indomethacin peaks in the nano-solid dispersion system are shifted (1679, 1614, 1590 and 1577 cm-1, respectively) and the spectrum appears simple. These results indicate that the crystalline structure of indomethacin in solid dispersions with PVP change to amorphous state and/or indomethacin molecules interact with carbonyl moiety of pyrrolidone ring of PVP by hydrogen bonds and make an eutectic mixture (21). These results are in agreement with earlier reports (9, 23).

#### Differential scanning calorimetry studies

**Figure 4:**
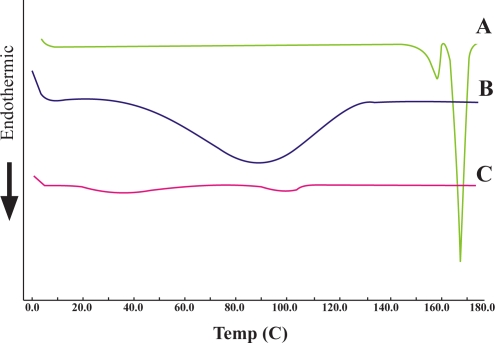
Differential scanning calorimeter thermograms of indomethacin (form I& II), PVP and nano-solid suspension (1:2).

Indomethacin interactions in nano-solid suspensions and physical mixture were investigated using DSC studies. The DSC curve of 1:1 ratio of pure indomethacin polymorphs exhibits two endothermic peaks with onset temperature of 148.15 °C and 158.83 °C respectively (Figure 4). It should be remembered that form II of indomethacin is a metastable form and re-crystallized to form I after melting at 148.15 °C. So, the endothermic picks are not equal. There are two negligible endothermic changes in the DSC curve of nano-solid suspension of indomethacin- PVP in the ratio of 1:2. Considering XRD pattern and FT-IR spectrum of nano-solid suspension one could presume that the indomethacin and PVP interact via hydrogen bond and forms a eutectic mixture. The first endothermic change appears as a board fluctuation in baseline with onset temperature at about 25 °C and the second as low broad peak with onset temperature at about 90 °C. These results suggest the formation of a eutectic system between the drug and polymer. In this case, the peak at the lower temperature belong to the melting of the eutectic mixture, and the second, peak which change at the higher temperature, indicate the melting of the surplus component. From the position of the second peak one could assume, that indomethacin is left over component after formation of eutectic mixture. To confirm this assumption a physical mixture composed of indomethacin and PVP in 2:3 ratio was scanned (data not shown). The peak of the eutectic appeared larger and the second peak was smaller than the DSC curve of lyophilized nanosuspensions with 2:1 ratio of components. These results demonstrate that the composition of the lyophilized sample is not in a eutectic ratio, but contains some excess of drug. (24). Therefore, the increase in the rate of dissolution of the drug can be explained by formation of the eutectic system and sub-micron sized drug particles during the production of nanosuspensions.

These data were supported with XRD studies. XRD patterns curves of PVP, indomethacin, physical mixture, and nano-solid suspension are shown in figure 2. Narrow and symmetrical peaks in indomethacin diffractogram (2*κ*=10–60°) indicate crystalline structural of this material. PVP on the contrary is dominantly amorphous in nature as indicated in its diffractogram. Evaluation of pure indomethacin, physical mixture and nano-solid suspension XRD diffractograms show that crystalline indomethacin (form I&II) undergo to amorphous state after dispread in solid state as freeze dried nanosuspensions.

#### Solubility and dissolution studies of lyophilized nano-solid suspensions

Indomethacin is a weak organic acid, with *p*K of 4.5 (25) and its solubility increases with pH. In order to simulate gastric condition for dissolution studies, a HCl (0.1 M) medium was selected to slow the dissolution rate of the drug, and as a result to allow greater discrimination of processing effects.

**Figure 5:**
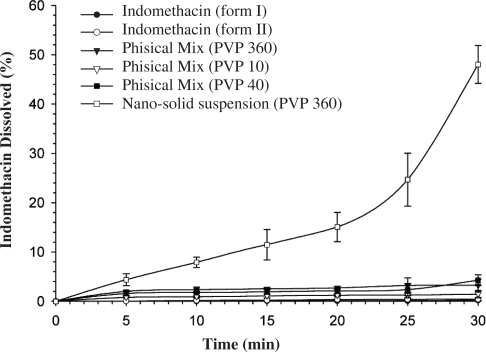
Dissolution profile of pure indomethacin (form I&II), physical mixtures and nano-solid dispersions, drug:polymer ratio (2:1).

The dissolution profiles of lyophilized indomethacin nano-solid suspensions, in comparison with a reference physical mixture of micronized indomethacin (Polymorph I & II) are shown in figure 5. The dissolution rate was markedly enhanced in the nanomatere-sized system, as more than 45 percent of drug dissolved in 30 min, as opposed to less than 10% of micronized drug in physical mixtures. This could be due to the increase in the surface area of the drug and possibly better wettability and dispersion of nano-solid suspensions in dissolution medium. In previous study, it was shown that the dissolution rate of nano-solid suspensions is dependent to polymer MW (14) and increased by decrease the polymer MW. In order to study the effect of PVP on solubility of indomethacin, intrinsic and relative solubility of indomethacin in nano-solid suspension and physical mixture of micronized indomethacin in comparison with forms I&II of indomethacin as a reference were investigated (Table 2). Intrinsic solubility of indomethacin in both physical mixture and solid dispersion in the presence of PVP significantly increased (*p < *0.05). This phenomenon can be explained by solubilization effects of PVP. Solubilization is likely to occur through the following mechanisms. In the dry state, drug particles were in close contact or adhered to the polymer particles as a result of mixing. When the mixture came in contact with water, the polymer particles might have hydrated rapidly into polymer solution, solubilizing the adjacent drug particles and subsequently releasing the drug into the medium (26). This could also possibly explain the higher solubility of drug in phase solubility study where the indomethacin particles were already dispersed in aqueous polymer solutions. Enhanced solubility and dissolution rate of indomethacin from physical mixtures could be possibly because of the combined action of the surface activity, solubilization and wetting effect of PVP (27–29).

**Table 2:** Comparison of intrinsic and relative solubility of indomethacin in different state.

Type system	Intrinsic solubility*	Relative solubility**
Nano-solid suspension	5.86±1.2	6.45
Physical mixture	2.30±0.51	2.53
Polymorph I	0.91±0.26	1.00
Polymorph II	1.44±0.34	1.59

* mg/100 ml of medium (n = 3)

** relative to polymorph I

It could be possible that high MW polymers (such as PVP K 360) in dissolution medium produce a viscose layer around nanoparticles released from freeze- dried nano-suspension and obstacle the molecular diffusion to dissolution medium. Therefore, a lag time in dissolution rate of indomethacin could be logical (13).

It has been reported that a progressive reduction in particle size corresponds to increases in the surface/volume ratio and the escaping tendency of the molecules until the nature of the surface dominates the properties of the material. According to Thomson-freundlich equation, saturation solubility of low soluble drugs is increased by decrease in particle size (30). However, this effect is only pronounced for particle below approximately 2 µm, especially with those of lower than 1 µm (31). So, reduction of the particle size of poorly soluble drugs such as indomethacin has an influent effect on dissolution rate, drug solubility and consequently bioavailability. The bioavailability of indomethacin is dependent to dissolution rate and particle size reduction can improve the drug efficacy (32).

## CONCLUSION

Nano-solid suspensions of indomethacin in PVP were prepared using a controlled pH co-precipitation method. Spectroscopic evidence clearly indicates that the drug molecules interactions with carbonyl groups of the polymer was dependent to polymer MW and drug ratios. The kinetics of drug release from the nano-solid suspension was superior to that of either indomethacin or the drug-PVP physical mixture, which was attributed to the simultaneous particle size reduction, the loss of crystallinity, and increased wettability due to the presence of the hydrophilic polymer.
